# Effects of Sexual Network Connectivity and Antimicrobial Drug Use on Antimicrobial Resistance in *Neisseria gonorrhoeae*

**DOI:** 10.3201/eid2407.172104

**Published:** 2018-07

**Authors:** Chris R. Kenyon, Ilan S. Schwartz

**Affiliations:** Instituut voor Tropische Geneeskunde, Antwerp, Belgium (C.R. Kenyon);; University of Cape Town, Cape Town, South Africa (C.R. Kenyon);; University of Alberta, Edmonton, Alberta, Canada (I.S. Schwartz)

**Keywords:** *Neisseria gonorrhoeae*, sexual networks, antimicrobial resistance, men who have sex with men, MSM, core groups, sexually transmitted infections, bacteria

## Abstract

Contemporary strategies to curtail the emergence of antimicrobial resistance in *Neisseria gonorrhoeae* include screening for and treating asymptomatic infections in high-prevalence populations in whom antimicrobial drug–resistant infections have typically emerged. We argue that antimicrobial resistance in these groups is driven by a combination of dense sexual network connectivity and antimicrobial drug exposure (for example, through screen-and-treat strategies for asymptomatic *N. gonorrhoeae* infection). Sexual network connectivity sustains a high-equilibrium prevalence of *N. gonorrhoeae* and increases likelihood of reinfection, whereas antimicrobial drug exposure results in selection pressure for reinfecting *N. gonorrhoeae* strains to acquire antimicrobial resistance genes from commensal pharyngeal or rectal flora. We propose study designs to test this hypothesis.

The rapid emergence of antimicrobial resistance (AMR) in *Neisseria gonorrhoeae* has led to fears that gonorrhea may soon become untreatable (*1*). An incompletely explained feature of the emergence of AMR in *N. gonorrhoeae* is its repeated emergence in core groups (*2*). As Lewis noted, AMR first emerged in core groups of sex workers in East Asia and elsewhere from the 1960s onward (*2*). In the past 3 decades, however, AMR has repeatedly emerged in men who have sex with men (MSM) (*2*,*3*). In both the United States and the United Kingdom, *N. gonorrhoeae* resistant to several antimicrobial drugs emerged in MSM years ahead of men who have sex with women (MSW) (Figure 1).

**Figure 1:**
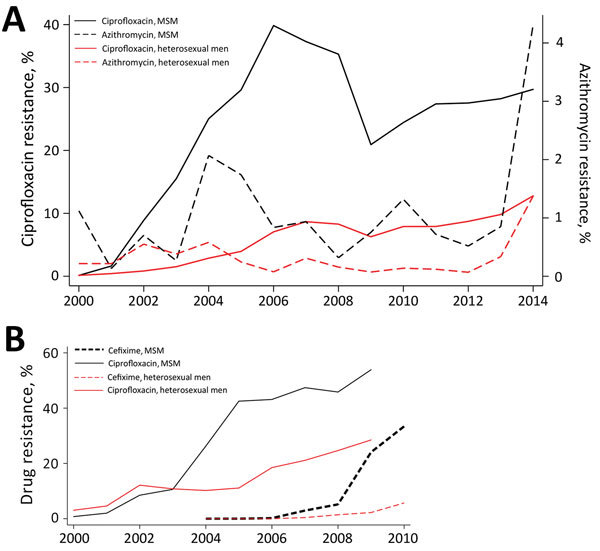
Prevalence of resistance to major antimicrobial drugs in *Neisseria gonorrhoeae*, United States and United Kingdom. A) Resistance to azithromycin and ciprofloxacin in the United States, 2000–2014. B) Resistance to cefixime and ciprofloxacin in the United Kingdom, 2000–2010. Data from Gonococcal Isolate Surveillance Program (USA) and Gonococcal Resistance to Antimicrobials Surveillance Programme (UK). MSM, men who have sex with men.

Several explanations have been proposed for this observation (*4*). Excess use of antimicrobial drugs is a possibility. One study found that MSM with a diagnosis of gonorrhea were more likely to report recent antimicrobial drug use than were MSW. After controlling for antimicrobial drug use, however, MSM remained at significantly higher risk for *N. gonorrhoeae* with resistance to all classes of antimicrobial drugs tested (p<0.001 for all) (*3*). Higher prevalence of HIV, which has been associated with various types of AMR in some studies, could also cause elevated resistance rates in MSM groups (*5*).

We focus on the emergence of *N. gonorrhoeae* AMR in MSM and hypothesize that the combination of high sexual network connectivity and excess antimicrobial drug use plays an important role in AMR genesis. This connectivity–AMR hypothesis proceeds in 2 steps: first, that high-equilibrium prevalence of *N. gonorrhoeae* in contemporary MSM populations is a function of densely connected sexual networks; and second, that extensive antimicrobial drug use (such as with STI screening and treatment) may temporarily reduce *N. gonorrhoeae* prevalence in this setting but produce selection pressure for *N. gonorrhoeae* to acquire AMR.

## STI Prevalence as a Function of Network Connectivity

STIs are transmitted along sexual networks, and as a result, the equilibrium prevalences of these STIs are determined by structural characteristics of these networks (*6*,*7*). These characteristics include the number of partners per unit time, prevalence of concurrent partnering, size of core groups, type of sex, size of sexual network, length of gaps between partnerships, degree and type of homophily (preference for partners with similarities to oneself), and relationships between core and noncore groups (*7*). Combinations of these attributes should result in higher network connectivity in some populations than in others (*6*,*7*). Studies have found a correlation between markers of network connectivity and the prevalence of various major STIs (*7*), including *N. gonorrhoeae* (*8*,*9*). STI prevalence can also be influenced by other risk factors that can affect the probability of transmission per contact (such as male circumcision, condom use, and presence of other STIs) or the duration of infectivity (such as STI early detection and treatment efficacy) (*10*). Although the relative contributions of these risk factors to STI prevalence vary considerably among populations, a consistent feature of contemporary STI epidemics in MSM populations in numerous countries is their association with dense sexual networks (*11*,*12*). For instance, nationally representative data from the United Kingdom, United States, and Australia reveal that MSM report considerably more sexual partners per unit of time than do heterosexual men (Table 1). An example of the prevalence of multiple partnering in MSM is given by >180,000 participants in the European Men Who Have Sex with Men Internet Survey (*13*); 67% of respondents reported a nonsteady partner in the past year, and 37.7% of respondents reported >10 partners in the past year. These high rates of partner change, combined with high rates of partner concurrency (*14*) and other determinants of network connectivity, translate into dense networks (*7*). Network connectivity is particularly dense in preexposure prophylaxis (PrEP) cohorts, in which the median number of sex partners typically exceeds 10 per 90 days. In the iPrEx study, for example, participants reported a mean of 18 partners (SD ± 35) in the preceding 90 days (*15*). The resulting dense network typically sustains equilibrium prevalences of both *N. gonorrhoeae* and *Chlamydia trachomatis* at >10% (*15*). In comparison, the prevalence of *N. gonorrhoeae* in the general heterosexual population in the United Kingdom is estimated at <0.1% and of *C. trachomatis* at 1.3% (*16*).

**Table 1:** Number of partners of MSM and heterosexual men from Australia, the United States, and the United Kingdom*

**Survey description**	Sexual orientation of participants	Mean no. lifetime sex partners (95% CI or SD)	Median no. lifetime sex partners (IQR)	Mean (95% CI) or median no. recent sex partners†	Median no. recent sex partners (IQR)†
**ASHR II‡**	MSM	143.1 (95.7–190.6)	22 (7–100)	6.8 (5.1–8.5)	1 (1–10)
	Heterosexual men	17.9 (17.1–18.7)	8	1.4 (1.3–1.4)	1
**NHANES§ **	MSM	26.9 (7.8)	22 (4–100)	NA	NA
	Heterosexual men	14.8 (1.6)	8 (3–20)	NA	NA
**NATSAL II¶**	MSM	NA	NA	24.1	4
	Heterosexual men	NA	NA	3.8	1

*ASHR II, Australian Study of Health and Relationships II; IQR, interquartile range; NA, not available; NATSAL, National Surveys of Sexual Attitudes and Lifestyles (United Kingdom); NHANES, National Health and Nutrition Examination Survey (United States).  †For ASHR and NHANES, recent refers to the previous 12 months; for NATSAL II, recent refers to the previous 5 years.  ‡ASHR II is a nationally representative sample of adults 16–59 y in Australia. Data were collected during 2012–2013 (n = 20,094). §NHANES is a nationally representative sample of civilian, noninstitutionalized adults 18–69 y in the United States. Data were collected during 2009–2012 (n = 13,374). ¶NATSAL is a national probability sample of adults 16–44 y in the United Kingdom. Data were collected during 2000 (n = 11,161).

## Connectivity–AMR Thesis—Combination of High Prevalence of Antimicrobial Use and Network Connectivity as Cause of Resistance 

In the absence of an antimicrobial selection pressure, we would not expect a high prevalence of *N. gonorrhoeae* to lead to AMR (*17*,*18*). Under such pressure, however, *N. gonorrhoeae* has developed AMR to each antimicrobial therapy introduced to treat infections, often within as few as 3 years (*1*,*19*,*20*). This effect is similar to the rapid development of AMR observed in a range of other bacteria (*21*). Individual-level studies have also found recent antimicrobial drug use to be a risk factor for AMR in *N. gonorrhoeae* (*3*,*22*,*23*). We would thus expect higher rates of antimicrobial drug use to be a risk factor for the emergence of AMR. Four mechanisms have been proposed to explain this antimicrobial drug–induced selection of AMR in bacteria for which, as for *N. gonorrhoeae*, horizontal gene transfer is a major mechanism of AMR acquisition (Table 2) (*1*,*24*). We argue that high network connectivity coupled with antimicrobial exposure constitutes an emergent fifth pathway to AMR in *N. gonorrhoeae*. 

**Table 2:** Four mechanisms whereby antimicrobial usage might select for antimicrobial resistance in *Neisseria gonorrhoeae* in a population

Mechanism	Description
Emergence of resistance during treatment	A large proportion of *N. gonorrhoeae* infections, particularly in MSM, are asymptomatic colonization of the pharynx, where the penetration of many antimicrobials is relatively poor. Because of this or other reasons for suboptimal therapy, a subpopulation of antimicrobial-resistant *N. gonorrhoeae* may emerge from treatment and may subsequently be transmitted to others.
Reduced transmission of susceptible strains	Treating patients with antimicrobial-sensitive *N. gonorrhoeae* reduces the probability of transmission to others, which in turn increases the probability that others will become infected with resistant *N. gonorrhoeae* strains.
Increased susceptibility to colonization	Eradicating a susceptible *N. gonorrhoeae* strain with treatment may enable infection by a new, resistant *N. gonorrhoeae* strain previously excluded through bacterial competition. This is possible mainly in high-transmission settings.
Increased density of resistant bacteria following treatment	If a person is infected with an antimicrobial-resistant *N. gonorrhoeae* strain, treatment may eradicate susceptible competing commensal microbes. Relieved of competition, the resistant *N. gonorrhoeae* strain could expand in the vacated niche.

*Based on Lipsitch et al. (*18*).

**Figure 2:**
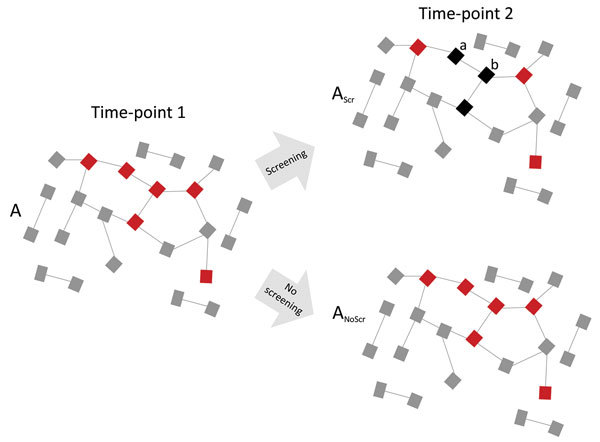
Diagram showing how high network connectivity combined with excess antimicrobial drug exposure from *Neisseria gonorrhoeae* preexposure prophylaxis could produce antimicrobial resistance (AMR). A dense sexual network translates into a high equilibrium prevalence of *N. gonorrhoeae* (red squares) at time point 1. Active *N. gonorrhoeae* screening of 50% of this population every 3 months results in 50% lower *N. gonorrhoeae* prevalence at time-point 2 (3 months later) but at the expense of an altered resistome (A_Scr_; black squares represent 3 patients with *N. gonorrhoeae* cleared by screening and treatment). The unchanged underlying network connectivity means that the prevalence of antimicrobial-sensitive *N. gonorrhoeae* is now 50% of its equilibrium prevalence, but if it acquired AMR it could return to its equilibrium prevalence. Furthermore, recently cured patients (a and b) are at high risk for reinfection from their partners at a time when their resistomes are enriched with resistance genes. Early reinfecting *N. gonorrhoeae* can acquire AMR by taking up these resistance genes by transformation. The screening program has thus both placed a selection pressure for the emergence of AMR and provided the resistance genes needed for AMR. In the absence of screening and excess antimicrobial drug use (A_NoScr_), *N. gonorrhoeae* prevalence would not decline, but there would be no pressure to select for antimicrobial resistance. Gray squares indicate uninfected persons; lines represent sexual relationships.

Figure 2 is a schematic representation of a PrEP MSM cohort with a dense sexual network and quarterly *N. gonorrhoeae* and *C. trachomatis* screening. In the absence of a global screen-and-treat strategy that leads to *N. gonorrhoeae* extinction, a typical local screen-and-treat approach induces a temporary decline in *N. gonorrhoeae* prevalence. Without altering the underlying determinant of high *N. gonorrhoeae* prevalence (network connectivity), *N. gonorrhoeae* tends to return to its high-equilibrium prevalence. Moreover, this strategy also increases the prevalence of the genes that encode AMR in *N. gonorrhoeae*. On the basis of studies using macrolides for other indications, ≈90% of patients treated with ceftriaxone and azithromycin, the currently recommended therapy for *N. gonorrhoeae* infection, would be expected to acquire macrolide resistance that can persist for up to 4 years in commensal pharyngeal and colonic bacteria (*25**,**26*). Recently treated patients are also at high risk for early reinfection because it is unlikely that their whole local sexual network has been effectively screened via partner tracing (*27*).

*N. gonorrhoeae* has a highly developed system of transformation to take up DNA from its environment, particularly from other *Neisseria* spp. (*28*), which, along with other mechanisms, may lead to AMR acquisition (*1*). Studies have established that transformation is a method by which *N. gonorrhoeae* acquired resistance to cefixime from commensal pharyngeal *Neisseria* species (*19*,*24*).

Further examples of the connectivity–AMR mechanism come from various branches of high-density animal husbandry in which antimicrobial drug–based strategies used alone to combat epidemics have led to the induction of AMR (*29*). Norwegian salmon farms, for example, contain roughly 200,000 salmon per pen (with population densities of <25 kg/m^3^) and consequently are prone to outbreaks of various bacterial, viral, and parasitic diseases (*29*,*30*). Initially, these epizootic infections were controlled predominantly with prophylactic and therapeutic antimicrobial drugs, but the bacterial and ectodermal pathogens rapidly developed resistance (*29*). Consequently, zoo sanitation (increased separation and fallowing of the fish) and vaccination were introduced, which allowed a decrease of antimicrobials used from 48 tons to 1 ton annually while reducing the number and severity of outbreaks and increasing the total salmon harvest (*29*). Studies from other sites have linked declines in antimicrobial drug use to declines in AMR in salmon-associated infections (*31*).

We acknowledge that there is conflicting evidence of whether an excess use of antimicrobial drugs results in AMR. A recent ecologic analysis from the United States, for example, found no association between antimicrobial prescribing and gonococcal AMR in 23 STI clinics (*32*).

## Effects of *N. gonorrhoeae* Screening on AMR

Theoretically, a sufficiently intense and synchronized global screen-and-treat program could lead to the extinction of *N. gonorrhoeae*. However, if the screening program falls short of complete eradication, and if a combination of high network connectivity and antimicrobial drug exposure is responsible for AMR, then paradoxically the more effective the screening program is at decreasing prevalence, the greater this AMR selection pressure would be. This conclusion is at odds with current initiatives to enhance *N. gonorrhoeae* screening in MSM and other high–*N. gonorrhoeae* prevalence populations. Screening for *N. gonorrhoeae* every 3–12 months is typically recommended in clinical guidelines for sexually active MSM (*33*). A notable exception to these guidelines is that from the US Preventive Service Task Force, which concluded that the absence of randomized controlled trials (RCTs) evaluating the merits of screening in men precluded recommendations on the matter (*34*). In MSM-PrEP programs, screening is recommended every 3–6 months (*35*). Longitudinal analyses of PrEP studies typically show high *N. gonorrhoeae* prevalences that do not decline despite frequent screening (*15*,*35*,*36*). A recent PrEP study found that the prevalence of *N. gonorrhoeae* remained static in the pharynx and rectum and increased in the urethra despite quarterly screening (*35*). Modeling studies have found that increasing screening intensity in MSM populations results in either a modest (*37*) or dramatic (*38*) reduction in *N. gonorrhoeae* prevalence.

Some authors have gone further and argued that screening is an important component for containing AMR emergence in *N. gonorrhoeae* (*2*,*20*). A recent paper on this topic for example outlined the argument as follows: “Gonococcal AMR will only be effectively mitigated when the global gonorrhea burden is reduced. Increased detection and effective treatment of asymptomatic gonorrhea in general and pharyngeal gonorrhea in particular are critical, because these infections are potential gonococcal reservoirs in which AMR (especially extended spectrum cephalosporin AMR) can emerge. Oropharyngeal infections are prevalent, mostly asymptomatic, and more difficult to treat; accordingly, screening and treatment in high-risk patients are important” (*20*). For similar reasons, the World Health Organization (WHO) has made the early detection and treatment of asymptomatic *N. gonorrhoeae* a key component of its plan to reduce the prevalence of *N. gonorrhoeae* infection by 90% by 2030 as well as *N. gonorrhoeae* AMR (*39*).

## Future Evaluation of the Connectivity–AMR Hypothesis

Increasing screening of high-risk patients to combat AMR is diametrically opposed to our connectivity–AMR thesis. Given the stakes involved (including untreatable infections), establishing the validity of the connectivity–AMR hypothesis in general and the place of screening in high-prevalence populations specifically is imperative. Part of the answer lies in accurately describing the mechanisms underpinning AMR in *N. gonorrhoeae* compared with other organisms. In some pathogens, such as *Mycobacterium tuberculosis,* resistance emerges primarily through mutations during treatment in hosts (*18*). For these pathogens, screening and treating infected persons is crucial for containing the spread of AMR (*18*). For other bacteria, such as *Streptococcus pneumonia*, *Enterococcus* spp., *Staphylococcus aureus*, and *N. gonorrhoeae*, horizontal gene transfer is the predominant means of acquisition of AMR (*40*). For these bacteria, AMR is driven predominantly by indirect population-level mechanisms of selection (Table 2) (*18*). Although screening for these organisms may reduce prevalence, it may also increase AMR by these indirect mechanisms. Two types of study could assess the connectivity–AMR thesis and the net benefits and harms of *N. gonorrhoeae* screening programs in MSM: RCTs and modeling studies. 

Community RCTs in high-connectivity populations, including MSM who are taking PrEP, could assess the effect of *N. gonorrhoeae* screening and treatment (vs. no screening and limiting therapy to patients with symptomatic *N. gonorrhoeae* infection) on several parameters: prevalence of *N. gonorrhoeae* infection; susceptibility to other STIs, including HIV; effect on adaptive immunity to *N. gonorrhoeae*; effect on individual and population resistome and microbiome; and emergence of AMR. Researchers could increase the probability of reducing *N. gonorrhoeae* prevalence in these studies by including aggressive contact tracing strategies, such as by using sexual networking apps. A practical challenge would be the large cohort size required to demonstrate a difference in the probability of AMR between the screening and no-screening arms of the study because AMR emergence is a rare event. Nonetheless, establishing whether *N. gonorrhoeae* screening reduces infection prevalence in dense networks and at what cost to the resistome (individual and population) would be informative. If screening is found to have little or no effect on *N. gonorrhoeae* prevalence but a large effect on the population resistome, it may cause a reevaluation of screening policies. Researchers could also assess the significance of altered resistomes to AMR in *N. gonorrhoeae* in vitro by assessing whether *N. gonorrhoeae* is able to acquire AMR via transformation with DNA extracts from posttreatment microbiomes. Such studies could also provide the probabilities of resistome alteration following specific therapies (including the decay curves of these alterations). These data could then be used to construct more realistic models of AMR induction in *N. gonorrhoeae* (*41*).

Recent modeling studies have found that screening high-connectivity MSM populations could reduce *N. gonorrhoeae* prevalence by ≈50% (*38*), but at the expense of an 11-fold increase in antimicrobial drug exposure (*37*). Few studies have evaluated the effect of screening on the emergence of AMR. One such study found evidence of a screening paradox: although screening the core group was crucial to reduce prevalence of *N. gonorrhoeae,* this strategy involved the highest risk of inducing AMR (*42*). However, that study used a compartmental model of the underlying sexual network and examined only 1 type of *N. gonorrhoeae* AMR, chromosomally mediated AMR (*42*). Future models that evaluate the probability of AMR emergence should use individual-based models that can model AMR via horizontal gene transfer. These models could assess if the combination of high connectivity and antimicrobial exposure more likely to produce and disseminate AMR than the combination of low connectivity and high antimicrobial exposure or of high connectivity and low antimicrobial exposure. Using models could also help establish the level of intensity required of screen-and-treat programs for highly connected sexual networks to reduce the prevalence of *N. gonorrhoeae* to a level with minimal risk for reinfection during the period when the resistomes of treated persons are altered. Our discussion has focused on MSM populations taking PrEP, but similar arguments would apply to other segments of the MSM sexual network, such as HIV-infected MSM who are excluded from PrEP programs and MSM without HIV infection who are not taking PrEP. Modeling studies could explore how differential network connectivity and antimicrobial drug exposure in different sections of MSM sexual networks may interact to produce AMR.

## Allodemics of Resistance in MSM

The connectivity–AMR theory makes 2 other predictions regarding AMR in MSM. The first is that *N. gonorrhoeae* will become resistant to the full range of antimicrobial drugs to which the population is exposed. In accordance with the connectivity–AMR theory, any widely used antimicrobial drug that reduces *N. gonorrhoeae* prevalence in MSM populations is at risk for AMR. Although not all studies have reached the same conclusion, the data from the national *N. gonorrhoeae* surveillance projects in the United States and United Kingdom have generally found this to be true (*3*,*43*). In the United States, for example, MSM were statistically more likely to have AMR *N. gonorrhoeae* for all classes of antimicrobial drugs tested (*3*).

The second prediction is that AMR in other bacterial STIs will be likely to emerge or to become more prevalent in MSM. Although the link is not as clearly established as with *N. gonorrhoeae*, there is some evidence that this is the case. For example, in the United States and Australia, macrolide resistance in *Treponema pallidum* first emerged in predominantly MSM populations at roughly the same time as azithromycin resistance in *N. gonorrhoeae* (*44*,*45*). In addition, the prevalence of macrolide resistance in *Mycoplasma genitalium* in MSM in 1 Australia study was found to be approximately double that of heterosexual men (*46*). Outbreaks have occurred in MSM of macrolide- or quinolone-resistant sexually transmissible enteric organisms *Shigella* spp. (*47*) and *Campylobacter* spp. (*48*). One phylogenetic analysis of *Shigella flexneri* infections from 29 countries concluded that the 3a serotype had emerged and acquired multiple AMR mutations while circulating sexually in international MSM sexual networks characterized by high rates of reinfection with this same serotype (*47*). Outbreaks of sexually transmitted methicillin-resistant *Staphylococcus aureus* have also been described in MSM (*49*).

**Figure 3:**
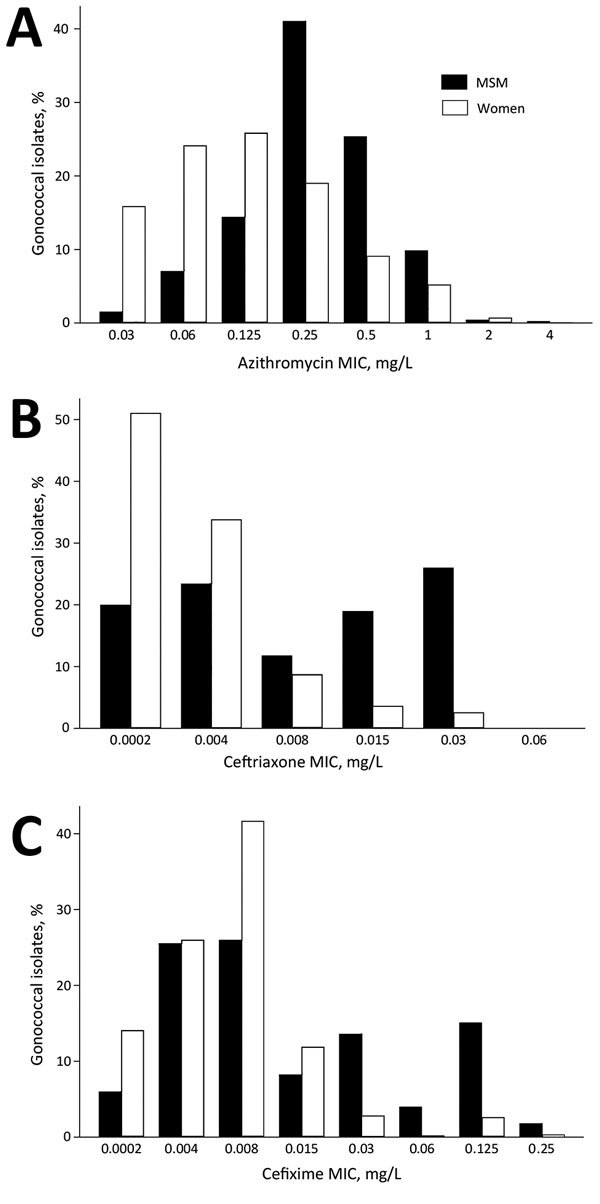
Comparison of distribution of drug MICs for *Neisseria gonorrhoeae* isolates from MSM and from women as determined by surveillance reports from the United Kingdom. A) Azithromycin, 2015; B) ceftriaxone, 2010; C) cefixime, 2011. Data from the Gonococcal Resistance to Antimicrobials Surveillance Programme. MSM, men who have sex with men.

**Figure 4:**
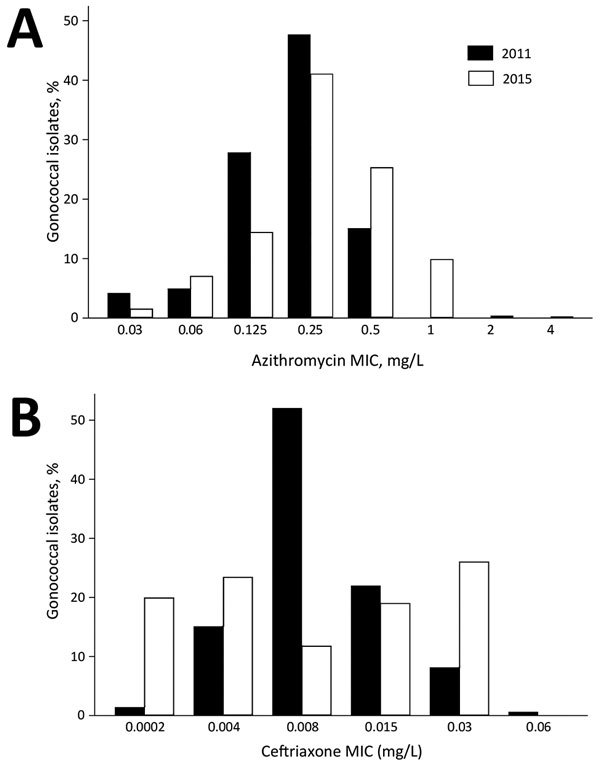
Comparison of distribution of drug MICs for *Neisseria gonorrhoeae* isolates by year as determined by surveillance reports from the United Kingdom. A) Azithromycin, 2011 and 2015; B) ceftriaxone, 2010 and 2015. Data from Gonococcal Resistance to Antimicrobials Surveillance Programme.

The variations in population shifts of *N. gonorrhoeae* MICs to various antimicrobial drugs by sexual orientation are also compatible with the connectivity–AMR thesis. The earliest available data for *N. gonorrhoeae* sensitivity by sexual orientation from the United Kingdom reveal that MSM have a higher proportion of *N. gonorrhoeae* with high MICs than women do (Figure 3). Furthermore, the evolution of *N. gonorrhoeae* MIC distributions in MSM from 2010–2015 reveals a right shifting of the whole distribution curve, indicating a reduction in the proportion of MSM with low MICs (Figure 4).

Considered together, these findings support the hypothesis that the problem of AMR in MSM could constructively be viewed as an allodemic of AMR. Baquero et al. first introduced this term to describe how the spread of extended spectrum β lactamase (ESBL)–producing bacteria in a hospital in Spain was best described by an increase in ESBL production in a range of bacteria (an allodemic) rather than epidemics of single species or clones (*50*). They argued that appreciating this polyclonal spread of resistance as an allodemic enabled them to address the underlying environmental determinant of AMR: excess use of antimicrobial drugs that induce ESBL production in multiple bacteria species rather than traditional approaches targeting individual clones or species (*50*). The problem of polyclonal AMR in MSM may likewise benefit from efforts to address the underpinning environmental determinants.

## Conclusions

Although we have focused our discussion on the connectivity–AMR thesis in MSM, similar considerations would also apply to other high connectivity populations. The emergence of *N. gonorrhoeae* AMR in sex workers, for example, has been linked to extensive antimicrobial drug use (*2*). Various studies have also concluded that high rates of STIs in various populations in sub-Saharan Africa are underpinned by dense sexual networks (*7*). In keeping with WHO directives, interventions are being planned to detect and treat asymptomatic STIs in South Africa and elsewhere. If the connectivity–AMR thesis applies to these populations, then due caution should be exercised if screening and antimicrobial drug use are used to reduce STI prevalence. In high-connectivity populations, particular consideration should be given to the use of nonantimicrobial STI therapies such as local disinfectants (e.g., for pharyngeal STIs), bacteriophage therapy, and vaccines. If antimicrobial drugs are used, research is required to guide their selection on the basis of efficacy and resistogenicity of therapies. Genotypic resistance profiling before therapy could also be considered. If STI prevention and control programs are unable to attain the level of screen-and-treat coverage required to eradicate STIs (or make negligible the risk for reinfection during the period of posttreatment resistome alteration), then they should prioritize STI reduction strategies that minimize the risk for AMR selection. These strategies would include methods to fragment sexual network connectivity (e.g., through decreasing rates of partner change) and treat STIs with nonantimicrobial therapies (e.g., bacteriophages and antiseptics).
